# Practical Notes and Formulæ

**Published:** 1888-11

**Authors:** 


### PRACTICAL NOTES AND FORMULÆ.

**To Abort Furunculus.**—Dr. Halle (*Prat. Med.*) recommends the following:

℞. Tr. arnica flowers.............................parts ij,
Tannic acid and powdered gum arabic......aa " j.

The mixture is to be painted upon the seat of the trouble and the surrounding parts every fifteen minutes until a thick and resistant coating is formed. The pain is immediately quieted and the furunculus aborted.

**Neuralgia.**—As a pomade for neuralgia the following has been recommended:

℞. Menthol....................................gr. xv,
Cocaine.......................................gr. v.
Chloral hydrat................................gr. iij,
Vaseline......................................ʒ j.

M.—*Coll. and Clin. Record.*

**Muriate of Ammonia in Myalgia.**—In a letter to the *St. Louis Medical and Surgical Journal,* Dr. Wm. Henry, of Harmon, Ills., calls attention to this old but neglected mode of treatment. He says: I have for a number of years used nothing but hydrochlorate of ammonia in these cases with almost uniform success. I give it in large doses—20 to 30 grains three or four times a day. The remedy should be kept up for some time after the pain has ceased.—*Medical Journal.*

**To Abort a Boil.**—Dr. Halie recommends the following application:

℞. Tinct arnicæ flor.............................parts ij,

Acid. Tannic.................................. " j,

Acaciæ Pulv................................... " j.

M. Paint upon the part and the surface immediately surrounding it every fifteen minutes, until a thick and resistant coat results.

This will speedily relieve the pain and abort the boil.

**Earache.**—A liniment is recommended by Paresi for this affection, composed of

℞. Camphorated Chloral.......................parts v,
Glycerine..................................... " xxx,
Oil of sweet almonds......................... " x.

It is applied twice daily on soft cotton, being introduced as far as possible into the ear, and may also be rubbed behind the ear. The pain is almost instantly relieved, and the inflammation in many cases is subdued. The liniment must be kept in carefully closed bottles.—*Medical Brief.*

**Bromidrosis.**—A dusting-powder consisting of—

℞. Boracic acid ................................ parts xv,
Oxide zinc ................................ " xxv,
French chalk ................................ " lx.

Applied freely to the socks as well as to the feet, gives relief. Another method is to apply boroglyceride after carefully drying and rubbing the feet. Special attention should be paid to the skin between the toes.—*Ib.*

**Treatment of Rheumatism.**—Peabody treats his cases of acute rheumatism with the following combination of salicylic acid and iron:

℞. Acid. salicylic ................................ gr. xx,
Ferri pyrophosphatis ................................ gr. v,
Sodii phosphatis ................................ gr. j,
Aquæ ................................ ℥ ss.

M. Sig.—The dose, which is described in this formulæ, is given every two hours.—*Ib.*

**Eczema of the Head.**—First carefully wash the head of the child with soap and water and then apply the following ointment:

℞. Acid. salicylic ................................ grs. xxv,

Tinct. benzoin ................................ ʒ j,

Vaseline ................................ ℥ j.

M.—*Ib.*

**Habitual Constipation.**—The best prescription for habitual constipation associated with hepatic torpor is:

℞. Chionia (Peacock) ................................ ℥ v,

Tinct. nucis vom ................................ ʒ ij,

Tinct. bellad. fol ................................ ʒ ij.

M. Sig.—Teaspoonful at bedtime in water. The dose can be diminished gradually.—*Ib.*

**Leucorrhœa.**—Two very stubborn cases of leucorrhœa, complicated or attended with asthma, yielded readily to the following:

℞. Ext. cereus blonblandii ................................ fʒ j,

Ext. pichi ................................ fʒ j,

Aqua, q. s. to ................................ ℥ iv.

M. A teaspoonful before each meal, or three times daily.—*Eclectic Medical Journal, Cincinnati.*

**Worms.**—A few months ago Dr. Locke prescribed for my little boy—

℞. Santonin ................................ gr. j,

Sugar of milk ................................ gr. vi.

M. Make six powders, take one powder before each meal. And it did its work too. Then I thought of the past with its horrors and its nastiness, of the present, in this instance, and its contrasting pleasantness.—*Lloyd.*

